# Multiple testing for gene sets from microarray experiments

**DOI:** 10.1186/1471-2105-12-209

**Published:** 2011-05-26

**Authors:** Insuk Sohn, Kouros Owzar, Johan Lim, Stephen L George, Stephanie Mackey Cushman, Sin-Ho Jung

**Affiliations:** 1 Biostatistics and Bioinformatics Center, Samsung Cancer Research Institute, Samsung Medical Center, Seoul, 137-710, Republic of Korea; 2 Department of Biostatistics and Bioinformatics, Duke University Medical Center, NC 27710, USA; 3 Department of Statistics, Seoul National University, Seoul 151-747, Republic of Korea; 4 Department of Medicine, Division of Medical Oncology, Duke University, NC 27710, USA

## Abstract

**Background:**

A key objective in many microarray association studies is the identification of individual genes associated with clinical outcome. It is often of additional interest to identify sets of genes, known a priori to have similar biologic function, associated with the outcome.

**Results:**

In this paper, we propose a general permutation-based framework for gene set testing that controls the false discovery rate (FDR) while accounting for the dependency among the genes within and across each gene set. The application of the proposed method is demonstrated using three public microarray data sets. The performance of our proposed method is contrasted to two other existing Gene Set Enrichment Analysis (GSEA) and Gene Set Analysis (GSA) methods.

**Conclusions:**

Our simulations show that the proposed method controls the FDR at the desired level. Through simulations and case studies, we observe that our method performs better than GSEA and GSA, especially when the number of prognostic gene sets is large.

## Background

One of the primary objectives in microarray association studies is the identification of individual genes that are associated with clinical endpoints such as disease type, toxicity or time to death. It is also of interest to examine the association between known biological categories or pathways and outcome. To this end, gene sets a priori believed to have similar biological functions from databases including KEGG [1] and Gene Ontology [2] are used. In recent years, a number of statistical methods have been proposed for the identification of significant genesets based on microarray experiments. Ackerman and Strimmer [3] list 36 methods, including [4-13], while outlining a general framework for formulating the hypothesis and analysis method for gene set inference.

In this paper, we propose a gene set analysis framework that utilizes classical theory for estimating equations to assess the association between each gene set and the outcome of interest. One of the statistical challenges in this setting is that there is dependency within each gene set, by virtue of coregulated genes belonging to the same gene set, as well as dependency across the gene sets since gene sets are not mutually exclusive. Our method will account for both intra-gene set and inter-gene set dependencies. Furthermore, given the large number of gene sets, one has to address the issue of multiple testing. The sampling distribution of our proposed procedure is approximated using permutation resampling to simultaneously address the dependency and multiple testing issues by controlling the false discovery rate (FDR; [14]). In the framework described by Ackerman and Strimmer [3], gene set analysis methods are broadly categorized as univariate or as global and multivariate procedures. Generally speaking, our method belongs to the latter category. The novelty of our proposed approach is that it leverages the flexibility of estimating equations to conduct inference for a variety of endpoints including binary, continuous, censored or longitudinal outcomes.

After presenting the theoretical and computational details for the proposed method, we summarize the results from a simulation study evaluating its statistical properties. We then apply the proposed method to analyze a number of microarray data sets. Finally, we provide a brief discussion to compare the performance of our method to those of two other methods: GSEA [6] and GSA [7]. For notational brevity, we will refer to transcripts on microarrays as genes, even though this may not be technically correct.

All analyses are carried out using the **R **statistical environment [15]. The code is available from http://www.duke.edu/~is29/GeneSet. Generalized inverses are computed using the pinv function from the maanova[16] extension package. The inverse of linear shrinkage covariance matrix is computed using invcov.shrink function in corpcor [17] extension package. The R extension packages R-GSEA[6] and GSA[18] are used to implement the GSEA and GSA methods respectively. The qvalue[19] extension package is used for calculating FDR adjusted P-values. For gene set and probe set annotation, Bioconductor [20] annotation packages (e.g., hu6800.db[21]) and Molecular Signature Database (MSigD; http://www.broad.mit.edu/gsea) annotation files are used.

## Methods

In these discussions, we will assume that RNA expression levels for *m *genes have been measured for *n *patients. Let us denote the set of genes on the microarray by *G *= {*G*_1_, ..., *G_m_*}. For patient *i*(= 1, ..., *n*), let *y_i _*denote the clinical outcome and *z_ij _*denote the measured gene expression level for *G_j_*. Let *G_j _*⊥ *Y *denote that expression of gene *j *is not associated with outcome. For each gene the marginal inference of interest will be canonically presented as testing *H_j _*: *G_j _*⊥ *Y *versus .

Suppose that for gene *j*, the hypotheses of independence can be quantified using a parameter say *θ_j_*. We assume that *θ_j _*= 0 indicates that *G_j _*and the outcome are independent. Thus, the hypotheses of interest can be expressed as testing *H_j _*: *θ_j _*≠ 0 against . We consider testing these marginal hypotheses within the context of general estimating functions, which for large *n*, are expressible in the form

where *U_ij_*(*θ_j_*) is a function of the data for subject *i *only so that *U*_1*j *_, ...,*U_nj _*are independent. The corresponding test statistic for *H_j _*will be *U_j_*(0). Let *μ_ij_*(*θ_j_*) = *E*(*U_ij_*) and . If *E*{*U_j_*(*θ*)} is a smooth function and *E*{*U_j_*(*θ*)} = 0 has a unique solution, then the solution  to *U_j_*(*θ*) = 0 is a consistent estimator of *θ_j_*. The family of score statistics [22] is a special case of this type of estimating equation.

A gene set is defined as a subset of *G*. We will assume that there are *K *pre-specified gene sets say  based on a given annotation database such as KEGG or GO. Note that  is usually a proper subset of *G *as not all genes are annotated. Let *m_k _*(*k *= 1, ..., *K*) denote the number of genes in gene set . We consider a gene set to be associated with the outcome of interest if at least one of its member genes is associated with the outcome. Let  denote that gene set  is not associated with the outcome *Y*. The hypotheses of interest from gene set k can then be denoted as testing  versus .

For notational convenience, for the remainder of this section we will focus on the first gene set  and assume that it consists of the first *m*_1 _genes, . Then the hypotheses of interest can be presented as testing  against . For testing this hypothesis, consider the vector , of the first *m*_1 _marginal statistics, which is approximately normal with marginal means *μ_j_*(*θ_j_*) and co-variances *σ*_*jj' *_(*j*, *j' *= 1, ..., *m*_1_). These quantities can be consistently estimated by  and

respectively, where . Let  and .

In the marginal testing setting, we have *μ_j_*(0) = 0 under *H_j_*, so we reject *H_j _*in favor of  if the realized value of  is large. The test statistic  has an asymptotic *χ*^2 ^distribution with 1 degree of freedom under the null distribution. For gene set  we will consider the test statistic

where . If *n *is large and *m*_1 _<*n*, the distribution of *W*_1 _under  is approximately *χ*^2 ^with *m*_1 _degrees of freedom. Similarly, we can compute ***U***_*k*_, *V_k _*and *W_k _*for any gene set .

In many cases, the sample size for a microarray study may not be large enough for the null sampling distribution to be well approximated by the theoretical limiting distribution. To address this issue, we propose calculating the *P*-values by approximating the exact null sampling distribution using permutation resampling. Note that the permutation distribution is generated under the hypothesis . That is, none of the *K *gene sets are associated with the outcome. This hypothesis is equivalent to the hypothesis ∩*_j_H_j _*(i.e., none of the genes are associated with outcome). Note that the latter intersection is restricted to G*, the set of annotated genes. A permutation replicate sample is obtained by randomly shuffling the the clinical outcomes {*y*_1_, ..., *y_n_*} while holding the gene expression matrix in place. This ensures that the intra-gene dependency structure is preserved while breaking the association between the genes and the outcome.

If *m_k _*is large, *V_k _*many not be reliably inverted numerically and, in the case where it exceeds *n*, is not invertible. For these cases, we consider the Moore-Penrose (MP) generalized inverse or the inverse of the linear shrinkage covariance matrix estimate *V*_LW _[13,23-25]. Here, we remark that the Hotelling's tests with the MP generalized inverse (g-inverse) and that with the inverse of *V*_LW _have been previously studied [13,23]. The MP g-inverse (of the sample covariance matrix) uses  to derive a test statistic , where *P_k _*is the eigen matrix and , ν_1_, ..., *ν_d _*are the *d *positive eigenvalues of *V_k_*. The asymptotic distribution of *W_k _*when *m_k _*is larger than *n *has been investigated extensively (e.g., [26-28]). The linear shrinkage estimate (LW) of *V *is *V*_LW _= *λV *+ (1 - *λ*)*E*, where *E *is a well conditioned target matrix and *λ *is the tuning parameter. The tuning parameter *λ *is chosen to minimize the Frobenius risk along with several candidates of target matrices [24,25].

### Two-Sample Tests

Suppose that there are two groups with *n_g _*subjects in group *g*(= 1, 2), *n *= *n*_1 _+ *n*_2_. Let ***z***_*gi *_= (*z*_*gi*1_, ..., *z_gim_*)^T ^denote the gene expression measurements from subject *i*(= 1, ..., *n_g_*) in group *g*(= 1, 2), and  the vector of sample means. Kong et al. [10] consider the Hotelling's *T*^2 ^statistic

where  is the pooled variance-covariance matrix. For *θ_j _*= *E*(*z*_1*ij*_) - *E*(*z*_2*ij*_) and *μ_ij_*(*θ_j_*) = *θ_j_*, *T*^2 ^asymptotically has a  distribution under *H*_0_.

For , our method gives

where  and . Since *T*^2 ^is asymptotically equivalent to *W *= ***U***^*T*^*V*^-1^***U ***under *H*_0_, we use the more popular Hotelling's *T*^2 ^statistic in this paper.

As a rank test alternative to the t-test, it is easy to show that the Wilcoxon rank sum test can be expressed as *T*^2 ^with *z_gij _*the rank of the gene *j *expression level for subject *i *in the pooled data {*z_gij_*, 1 ≤ *i *≤ *n_g_*, *g *= 1, 2}. In this case, *θ_j _*= *P*(*z*_1*ij *_≥ *z*_2*ij*_) - 1/2 and *μ_ij_*(*θ_j_*) = *θ_j_*.

### Linear Regression Case

Suppose that we want to relate the gene expression *z_ij _*for gene *j *with a continuous outcome *y_i _*through a linear regression

No association between *y *and the expression of gene *j *implies that *θ_j _*= 0. In this case, we use , the least square estimator of the slope *θ_j_*,

and

where ,  and .

### Cox Regression Case

For right-censored time to event data, the outcome data are pairs of the form *y_i _*= (*t_i_*, *δ_i_*), where *t_i _*is the minimum of survival and censoring times, and *δ_i _*is the event indicator. Let *λ_i_*(*t*) denote the hazard function of patient *i*. Then the Cox proportional hazards model relates the expression of gene *j*, *z_ij_*, with the survival time of patient i using the model *λ_ij_*(*t*) = *λ*_0*j*_(*t*) exp(*θ_j _z_ij_*), where *λ*_0*j*_(*t*) is an unknown baseline hazard function. We propose using the partial score statistic [29]*U_j _*= *U_j_*(0), where

and , *Y_i_*(*t*) = *I*(*t_i _*≥ *t*) and *N_i_*(*t*) = *δ_I_I*(*t_i _*≤ *t*). Let  denote the partial MLE of *θ_j _*solving the partial score equation *U_j_*(*θ*) = 0. In this case, we have

where

The resulting variance estimator is equivalent to the robust estimator under the possible violation of the proportional hazards model proposed by Lin and Wei [30].

## Results

### Simulation Study

We investigate the performance of our proposed method with respect to FDR control through a simulation study. Let *z_ijk _*denote the expression level of gene *j*(= 1, ..., *m_k_*) from subject *i *(= 1, ..., *n*_1 _+ *n*_2_) in the group *g*(= 1, 2) for gene set . We consider the following model:

where *s_i _*= 0 if subject i belongs to group 1 and *s_i _*= 1 otherwise, and *ρ*_3 _= 1 - *ρ*_1 _- *ρ*_2_. For gene *j*, *δ_j _*is the treatment effect, *D *is the number of prognostic genes, *K*_1 _is the number of prognostic gene sets, *a_k _*is the gene set effect, *b *is the array effect, *ρ*_1 _and *ρ*_2 _are the within gene sets and within arrays correlation coefficients resepctively, and *ε_ijk _*is the error term. The gene set effect *a_k_*, the array effect *b*, and the error term *ε_ijk _*are generated from independently and identically distributed *N*(0, 1) random variate.

At first, we investigate the performance of the test statistic using the MP inverse generalized inverse. We consider *m *= 1, 000 genes and *n *= 100 samples, each with non-overlapping *K *= 50 or 20 gene sets of *m_k _*= 20 or 50 genes, respectively, (*ρ*_1_, *ρ*_2_) = (0, 0), (0.2, 0.2) or (0.4, 0.4), *D*/*m_k _*= 0.2, 0.5 or 0.8, *δ *= 0.4 and *K*_1 _= 1 or 5. We conduct *N *= 1, 000 simulations under each setting, and approximate the null distribution of the test statistic using *B *= 10, 000 random permutations for each simulation. The *q*-values [31] are obtained from the resulting unadjusted permutation *P*-values by setting *λ *= 0.5. Results are presented in Table 1 where  denotes the empirical FDR and  denotes the mean number of true rejections, i.e. the mean number of prognostic gene sets that are discovered by testing. These results illustrate that the proposed method accurately controls the FDR at the desired level q*. The observed true rejection rate is high when the proportion of prognostic genes within each gene set is large (i.e., *D*/*m_k _*= 0.8).

**Table 1:** Empirical FDR and mean true rejections

				q* = 0.01		q* = 0.05		q* = 0.1		q* = 0.2	
(*m_k_*, *K*)	*K*_1_	*D*/*m*	(*ρ*_1_, *ρ*_2_)								
(50,20)	1	0.2	(0, 0)	0.010	0.10	0.063	0.25	0.105	0.35	0.189	0.46
			(0.2, 0.2)	0.015	0.08	0.053	0.28	0.128	0.38	0.231	0.45
			(0.4, 0.4)	0.013	0.12	0.045	0.25	0.087	0.36	0.215	0.47
		0.5	(0, 0)	0.011	0.72	0.060	0.88	0.107	0.95	0.220	0.98
			(0.2, 0.2)	0.015	0.71	0.051	0.89	0.122	0.94	0.212	0.97
			(0.4, 0.4)	0.005	0.74	0.071	0.89	0.115	0.94	0.266	0.95
		0.8	(0, 0)	0.013	0.97	0.057	1.00	0.106	1.00	0.215	1.00
			(0.2, 0.2)	0.013	0.98	0.065	1.00	0.138	1.00	0.235	1.00
			(0.4, 0.4)	0.018	0.97	0.078	1.00	0.124	1.00	0.253	1.00
	5	0.2	(0, 0)	0.025	0.72	0.067	1.70	0.136	2.51	0.239	3.56
			(0.2, 0.2)	0.009	0.68	0.056	1.79	0.127	2.63	0.235	3.59
			(0.4, 0.4)	0.011	0.74	0.058	1.82	0.118	2.46	0.232	3.55
		0.5	(0, 0)	0.007	4.44	0.058	4.94	0.124	4.97	0.227	5.00
			(0.2, 0.2)	0.013	4.46	0.056	4.90	0.118	4.97	0.214	4.99
			(0.4, 0.4)	0.012	4.44	0.074	4.87	0.137	4.96	0.239	5.00
		0.8	(0, 0)	0.012	4.99	0.066	5.00	0.120	5.00	0.235	5.00
			(0.2, 0.2)	0.011	5.00	0.056	5.00	0.105	5.00	0.200	5.00
			(0.4, 0.4)	0.011	4.98	0.053	5.00	0.101	5.00	0.218	5.00
(20,50)	1	0.2	(0, 0)	0.008	0.04	0.055	0.13	0.092	0.21	0.185	0.25
			(0.2, 0.2)	0.015	0.04	0.051	0.10	0.128	0.15	0.219	0.21
			(0.4, 0.4)	0.005	0.06	0.050	0.10	0.110	0.14	0.198	0.22
		0.5	(0, 0)	0.013	0.45	0.048	0.63	0.118	0.72	0.240	0.82
			(0.2, 0.2)	0.010	0.43	0.077	0.64	0.122	0.72	0.223	0.80
			(0.4, 0.4)	0.010	0.49	0.072	0.68	0.112	0.76	0.214	0.83
		0.8	(0, 0)	0.010	0.86	0.043	0.97	0.113	0.99	0.222	0.99
			(0.2, 0.2)	0.015	0.89	0.048	0.98	0.115	0.98	0.208	0.98
			(0.4, 0.4)	0.013	0.86	0.052	0.96	0.102	0.99	0.201	1.00
	5	0.2	(0, 0)	0.013	0.31	0.054	0.76	0.121	1.08	0.210	1.64
			(0.2, 0.2)	0.010	0.28	0.039	0.57	0.102	0.89	0.224	1.56
			(0.4, 0.4)	0.015	0.18	0.062	0.57	0.103	0.94	0.195	1.59
		0.5	(0, 0)	0.011	3.03	0.055	4.03	0.107	4.43	0.201	4.75
			(0.2, 0.2)	0.008	3.01	0.054	4.11	0.104	4.44	0.218	4.77
			(0.4, 0.4)	0.016	3.22	0.058	4.16	0.103	4.50	0.203	4.72
		0.8	(0, 0)	0.010	4.74	0.054	4.91	0.112	4.95	0.224	4.99
			(0.2, 0.2)	0.011	4.76	0.054	4.94	0.111	4.97	0.212	4.98
			(0.4, 0.4)	0.012	4.73	0.054	4.93	0.110	4.96	0.201	4.99

We proceed by investigating the case with small *n *but large *m_k_*. We set the sample size *n *= 20, and consider *K *= 20 and *m_k _*= 50. All other parameters are identical to those used in the simulation study reported in Table 1. We conduct *N *= 500 simulations and apply the test using both MP and LW generalized inverses. The results reported in Table 2 show that both tests control the FDR at the desired level q*. Similar to the results presented in Table 1, for both tests the observed true-rejection rate () increases in the proportion of prognostic genes within each gene set (*D*/*m_k_*). However, the test with the LW inverse has generally higher true-rejection rate than that with the MP generalized inverse.

**Table 2:** Empirical FDR and mean true rejections on simulation data with small *n *large *p *values.

				q* = 0.01		q* = 0.05		q* = 0.1		q* = 0.2	
*K*_1_	*D*/*m_k_*	(*ρ*_1_, *ρ*_2_)	method								
1	0.2	(0, 0)	MP	0.0160	0.004	0.0633	0.018	0.1102	0.036	0.2328	0.058
			LW	0.0060	0.018	0.523	0.040	0.1022	0.060	0.2294	0.098
		(0.2 0.2)	MP	0.0100	0.006	0.0593	0.010	0.1145	0.022	0.2325	0.042
			LW	0.0180	0.006	0.0563	0.020	0.1058	0.036	0.2249	0.084
		(0.4 0.4)	MP	0.0180	0.008	0.0590	0.020	0.1133	0.032	0.2182	0.054
			LW	0.0100	0.010	0.0503	0.032	0.1087	0.048	0.2387	0.094
	0.5	(0, 0)	MP	0.0120	0.024	0.0650	0.068	0.1138	0.100	0.2016	0.162
			LW	0.0210	0.078	0.0652	0.160	0.1189	0.230	0.2303	0.330
		(0.2 0.2)	MP	0.0300	0.024	0.0720	0.072	0.1269	0.106	0.2608	0.170
			LW	0.0200	0.084	0.0823	0.154	0.1467	0.222	0.2658	0.316
		(0.4 0.4)	MP	0.0290	0.038	0.0920	0.072	0.1359	0.112	0.2161	0.184
			LW	0.0150	0.070	0.0747	0.168	0.1300	0.228	0.2444	0.338
	0.8	(0, 0)	MP	0.0150	0.084	0.0667	0.136	0.1261	0.178	0.2534	0.258
			LW	0.0070	0.240	0.0520	0.404	0.1199	0.508	0.2179	0.618
		(0.2 0.2)	MP	0.0260	0.068	0.0563	0.142	0.1147	0.198	0.2341	0.276
			LW	0.0220	0.234	0.0603	0.420	0.1054	0.516	0.2095	0.622
		(0.4 0.4)	MP	0.0153	0.068	0.0738	0.138	0.1299	0.176	0.2548	0.258
			LW	0.0270	0.274	0.0607	0.434	0.1129	0.512	0.2118	0.636
5	0.2	(0, 0)	MP	0.0080	0.022	0.0503	0.094	0.0924	0.194	0.1817	0.386
			LW	0.0120	0.066	0.0443	0.188	0.0933	0.344	0.1883	0.726
		(0.2 0.2)	MP	0.0180	0.024	0.0683	0.084	0.1030	0.138	0.2140	0.364
			LW	0.0170	0.054	0.0642	0.144	0.1028	0.288	0.2125	0.646
		(0.4 0.4)	MP	0.0060	0.040	0.0350	0.078	0.0848	0.158	0.1969	0.304
			LW	0.0150	0.072	0.0446	0.192	0.0762	0.328	0.1761	0.668
	0.5	(0, 0)	MP	0.0143	0.158	0.0479	0.402	0.0990	0.608	0.2144	1.062
			LW	0.0140	0.442	0.0626	1.148	0.1221	1.734	0.2131	2.586
		(0.2 0.2)	MP	0.0157	0.148	0.0586	0.418	0.0974	0.662	0.2090	1.128
			LW	0.0127	0.452	0.0646	1.162	0.1076	1.796	0.2165	2.644
		(0.4 0.4)	MP	0.0160	0.150	0.0602	0.418	0.0978	0.678	0.2062	1.142
			LW	0.0108	0.498	0.0572	1.154	0.1134	1.680	0.2226	2.548
	0.8	(0, 0)	MP	0.0193	0.468	0.0662	1.006	0.1194	1.452	0.2320	2.054
			LW	0.0216	1.660	0.0609	3.044	0.1220	3.702	0.2385	4.286
		(0.2 0.2)	MP	0.0127	0.478	0.0567	1.036	0.1130	1.440	0.2257	2.156
			LW	0.0145	1.654	0.0506	2.970	0.1157	3.670	0.2269	4.278
		(0.4 0.4)	MP	0.0200	0.510	0.0587	1.020	0.1080	1.468	0.2163	2.164
			LW	0.0201	1.772	0.0729	3.066	0.1303	3.662	0.2350	4.234

Here, *n *= 20, *m_k _*= 50, and *K *= 20.

We compare the performance of our method to GSEA and GSA within the simulation framework described above. We choose, for GSEA, the weighted Kolmogorov Smirnov-like statistic as enrichment correlation-based weighting, while for GSA we choose the maxmean statistic along with restandardization. The technical details are provided in [6] and in [7] respectively.

We generate *m *= 1, 000 genes and *n *= 100 samples, each with non-overlapping *K *= 50 gene sets of *m_k _*= 20 genes, (*ρ*_1_, *ρ*_2_) = (0, 0), *D*/*m_k_*= 1, and *δ *= 0.4 as in [7]. The first (*n*_1 _= 50) and second (*n*_2 _= 50) samples will constitute the control and treatment groups respectively. Next, we will discuss two scenarios similar to those considered by [7]:

• One-sided shifts: The mean expression level for the *m_k _*= 20 genes in each of the *K*_1 _prognostic gene sets is *δ *= 0.4 units higher in the treatment group.

• Two-sided shifts: The mean expression level for the first 10 genes in each of the *K*_1 _prognostic gene sets is *δ *= 0.4 units higher, while the mean expression level for the next 10 genes is *δ *= 0.4 units lower.

**Figure 1:**
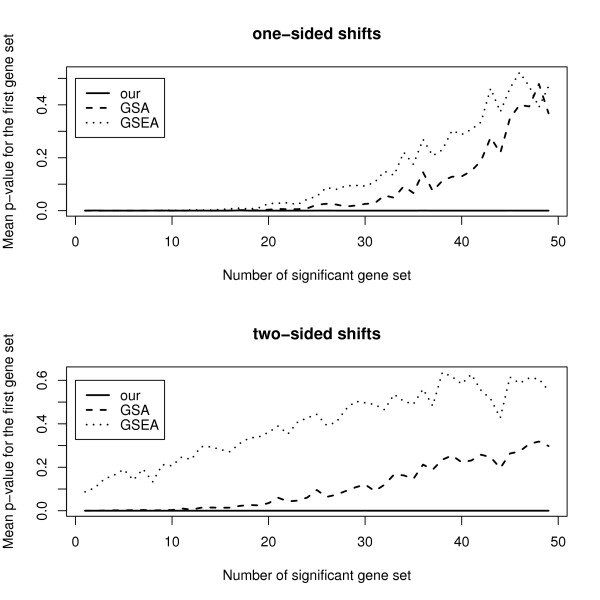
**Mean *P*-value against the number of prognostic gene sets**.

Each scenario is simulated 100 times using 1000 permutation replicates. The *P*-values for the first gene set is shown against the number of prognostic gene sets in Figure 1. Overall, our method gives lower mean *P*-values under both scenarios. In the one-sided shift case, the three methods are comparable when the number of prognostic gene sets is at most thirteen. For the cases with a large number of prognostic gene sets or a two-sided shift, our method is consistently better.

### Case Studies

#### Two-Sample Case

**Figure 2:**
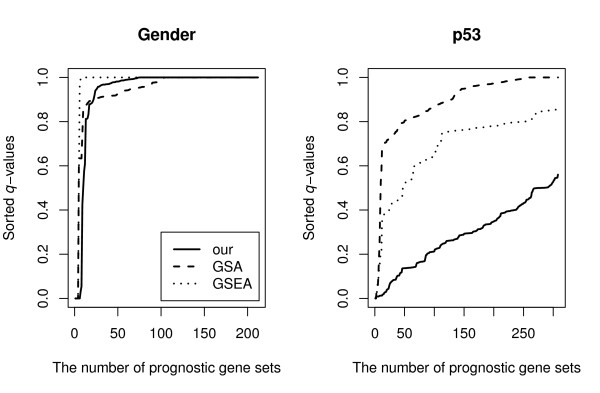
**The number of prognostic gene sets, at a given *q*-value threshold, identified by all three methods are shown for the Gender and p53 data sets**.

We analyze two microarray data sets available from the GSEA website http://www.broad.mit.edu/gsea. The first data set, called the Gender data set, consists of profiles of *m *= 15, 056 genes from male (*n*_1 _= 15) and female (*n*_2 _= 17) lymphoblastoid cell lines. The second data set consists of transcriptional pro-les of *m *= 10, 100 genes from p53 positive (*n*_1 _= 17) and p53 mutant (*n*_2 _= 33) cancer cell lines. The pathways from MSigDB are currently organized into five catalogs. We use the Positional gene sets (*C*_1_, 319 gene sets), which correspond to each human chromosome and each cytogentic band, for the Gender data set and the Curated gene sets (*C*_2_, 522 gene sets), which are derived from online pathway databases and publications, for the p53 data set. For the analyses, we limit our attention to gene sets which consist of a minimum of 15 and a maximum of 500 genes. Each analysis is based on 10,000 permutation replicates. The performance of our method is compared to those of GSEA and GSA. We compare the number of prognostic gene sets identified by each method. The analysis results for the two data sets are shown in Figure 2. For both data sets, our method consistently identifies more prognostic gene sets than GSEA and GSA for any q-value threshold.

#### Cox Regression Case

**Figure 3:**
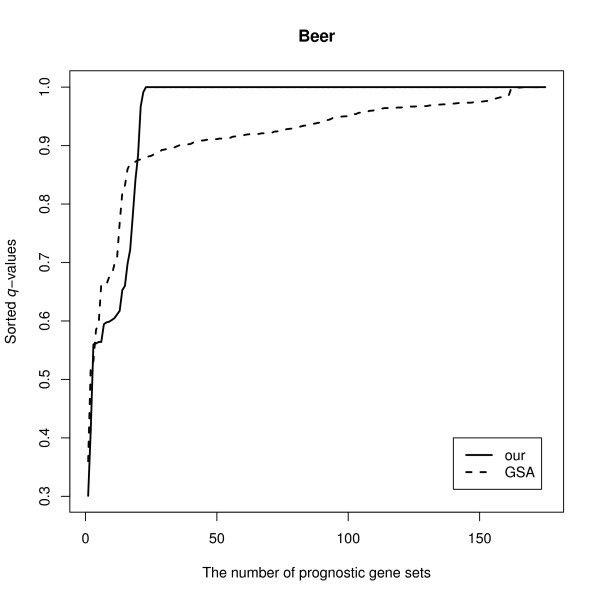
**The number of prognostic gene sets, at a given *q*-value threshold, identified by our and the GSA method are shown for the Beer Lung Cancer data set**.

We carry out gene set analysis of the lung cancer microarray data set [32] using the KEGG pathway (175 gene sets) provided by the hu6800.db Bioconductor package. The data set consists of gene expressions of m = 4, 966 genes from *n *= 86 stage I or III lung cancer patients. As in the analyses for the previous data sets, we include gene sets consisting of 15 to 500 genes each in the analysis, and use 10,000 permutations to derive the null distribution of the test statistics. For this analysis, we will compare our method to GSA only since the R-GSEA extension package does not provide the functionality for analyzing right censored data. The results are shown in Figure 3 suggest that our method generally identifies a larger number of prognostic gene sets compared to GSA.

## Discussion

For the Gender data set, at the FDR level of q* = 0.2, our method identifies 8 gene sets compared to only 4 for the other two methods [see Additional file 1]. There are 4 prognostic gene sets identified in common among the three methods, consisting of gene sets found on ChrY, ChrYp11, ChrYq11, and ChrXp22. Our method identifies 4 other gene sets not identified by the other two methods, which include gene sets for ChrX, ChrXp11, Chr3q25, and Chr6q25. Genes expressed on the Y chromosome are expected to be differentially expressed between genders, while gene expression from the X chromosome is more similar between genders due to X chromosome inactivation in females [33,34]. However, ChrXp22 and ChrXp11 gene sets have been previously been shown to be overrepresented in females likely caused by escape of X inactivation [35]. Furthermore, several genes within the Chr3q25 and Chr6q25 gene sets have also been shown to be differentially expressed between males and females, including ACAT2 [36], MAP3K4 [37], NOX, PTX3 [38], SGEF, and SOD2 [39]. Thus, our method for identifying overrepresented genes in gene set lists can provide biologically relevant and important information that may be overlooked by other common methods such as GSA and GSEA.

For the p53 data set, at the same FDR level, our method identifies 87 prognostic gene sets while GSA and GSEA identify 5 and 9 prognostic gene sets, respectively [see Additional file 1]. There are 5 prognostic gene sets common among the three methods, including the p53 pathway, hsp27 pathway, radiation sensitivity pathway, ceramide pathway, and the ras pathway. However, our method identifies 78 gene sets not identified by the other two methods. Additional file 1 also provides a list of gene sets that are identified only by our method. p53 is a tumor suppressor protein that is activated in response to DNA damage. p53 can induce growth arrest by halting the cell cycle at the G1/S phase transition to allow DNA repair or it can induce apoptosis if the DNA damage cannot be repaired. p53 acts as a transcription factor regulating the expression of many genes involved in its functions [40]. Thus many of the gene sets identified by our method can be directly linked to p53 functions, such as cell cycle arrest, ATM pathway, tumor suppressor, bcl2 family and network, death pathway, etc [40]. Additionally, several cytokine and growth factor signaling pathways are represented in our list of gene sets differentially expressed between p53 positive and mutant cell lines, including the IL-4 [41], EGF [42], NGF [43], CXCR4 [44], IL-7 [45], and PDGF [46] pathways, which have all shown roles for p53 in their regulation and signaling. The method that we describe here for identifying prognostic gene sets can provide a more inclusive list of gene sets that provide further insight into the biology of two sample case studies from microarray experiments.

## Conclusion

In this paper, we have presented a multiple testing procedure to identify prognostic gene sets from a microarray experiment correlated with common types of binary, continuous and time to event clinical outcomes. We calculate the marginal *P*-values using a permutation method accounting for dependency among the genes within and across each gene set, and account for multiple testing by controlling the FDR. Our simulations show that our proposed method controls the FDR at the desired level. Through extensive simulations and real case studies, we observe that our method performs better than GSEA and GSA, especially when the number of prognostic gene sets is large.

## Authors' contributions

IS and KO performed statistical analysis and wrote the manuscript. JL supported technical aspects of the research. SC provided biological interpretation of the gene sets found to be significant by the proposed method. SHJ and SLG proposed the research project. SHJ developed the methodological framework. All authors read and approved the final manuscript.

## Supplementary Material

Additional file 1 **Results from gene set analyses for the Gender and p53 data sets**. This file contains two tables. Tables 1 and 2 summarize gene set analysis results based on three methods for the Gender and p53 data sets respectively. Click here for file
