# Supplementary material for: Multiple testing for gene sets from microarray experiments
Source: BMC Bioinformatics. 2011 May 26;12:209. doi: 10.1186/1471-2105-12-209 (PMC3131260; doi:10.1186/1471-2105-12-209)
Supplement: Additional file 1 — Results from gene set analyses for the Gender and p53 data sets. This file contains two tables. Tables 1 and 2 summarize gene set analysis results based on three methods for the Gender and p53 data sets respectively. [file 1471-2105-12-209-S1.PDF]

Table 1. A list of gene sets selected by gene set methods on Gender

		P-value			Q-value		
Gene set	Size	Our	GSA	GSEA	Our	GSA	GSEA
chr6q25	40	0.0000	0.1965	0.5055	0.0000	0.9976	1.0000
chrX	409	0.0000	0.0160	0.0484	0.0000	0.6336	1.0000
chrY	40	0.0000	0.0000	0.0000	0.0000	0.0000	0.0000
chrYp11	18	0.0000	0.0000	0.0000	0.0000	0.0000	0.0000
chrYq11	16	0.0000	0.0000	0.0000	0.0000	0.0000	0.0000
chrXp22	76	0.0000	0.0000	0.0000	0.0000	0.0000	0.0000
chr3q25	26	0.0010	0.2835	0.4996	0.0311	1.0000	1.0000
chrXp11	95	0.0020	0.0075	0.0117	0.0545	0.7425	0.5644

Table 2. A list of gene sets selected by gene set methods on P53

		P-value			Q-value		
Gene set	Size	Our	GSA	GSEA	Our	GSA	GSEA
p53Pathway	16	0.0000	0.0000	0.0002	0.0000	0.0000	0.0151
atmPathway	19	0.0003	0.1894	0.2706	0.0087	1.0000	0.6988
calcineurinPathway	18	0.0002	0.2842	0.2282	0.0087	0.9391	0.6390
hsp27Pathway	15	0.0002	0.0008	0.0000	0.0087	0.0486	0.0000
raccycdPathway	22	0.0003	0.1299	0.6667	0.0087	1.0000	0.7882
radiation_sensitivity	26	0.0002	0.0000	0.0038	0.0087	0.0000	0.1450
g1Pathway	26	0.0004	0.1554	0.4944	0.0099	1.0000	0.7558
cellcyclePathway	23	0.0007	0.1505	0.7513	0.0122	1.0000	0.8026

drug_resistance_and_metabolism	95	0.0007	0.0718	0.2163	0.0122	0.9490	0.6253
cell_cycle_regulator	23	0.0008	0.0150	0.3672	0.0127	0.5700	0.7630
cell_cycle_arrest	30	0.0006	0.1470	0.4280	0.0131	0.8190	0.7745
SA_TRKA_RECEPTOR	16	0.0009	0.0642	0.2723	0.0131	0.7704	0.6898
mitochondriaPathway	19	0.0013	0.2137	0.3938	0.0174	0.8231	0.7759
cell_proliferation	200	0.0015	0.3583	0.8975	0.0186	0.9902	0.8496
ceramidePathway	22	0.0017	0.1735	0.4794	0.0197	1.0000	0.7591
p53hypoxiaPathway	20	0.0024	0.0004	0.0000	0.0261	0.0405	0.0000
g2Pathway	23	0.0029	0.0702	0.5432	0.0280	0.7065	0.7645
badPathway	21	0.0028	0.0051	0.0625	0.0287	0.2584	0.4491
gleevecPathway	22	0.0033	0.1019	0.1686	0.0302	0.7570	0.6142
PGC	315	0.0044	0.3543	0.9531	0.0383	0.8987	0.8451
chemicalPathway	21	0.0052	0.3882	0.0627	0.0411	0.9917	0.4386
MAP00562_Inositol_phosphate_metabolism	18	0.0051	0.1421	0.3327	0.0423	1.0000	0.7563
p53_signalling	87	0.0058	0.0303	0.0596	0.0439	0.9211	0.4407
ck1Pathway	15	0.0076	0.0122	0.0322	0.0551	0.5298	0.4082
ca_nf_at_signalling	95	0.0092	0.0330	0.1053	0.0640	0.8360	0.5384
tRNA_Synthetases	17	0.0110	0.3524	0.7400	0.0709	0.9087	0.7970
mitochondr	330	0.0108	0.4661	0.8382	0.0723	0.9446	0.8289
ST_Dictyostelium_discoideum_cAMP_Chemotaxis_Pathway	31	0.0128	0.0969	0.0796	0.0768	0.7374	0.4600
tnf_and_fas_network	20	0.0125	0.0690	0.4166	0.0777	0.7176	0.7749
NFKB_INDUCED	106	0.0140	0.4994	0.9328	0.0812	1.0000	0.8411
MAPK_Cascade	21	0.0167	0.0136	0.0065	0.0830	0.8486	0.1912
deathPathway	31	0.0154	0.1700	0.2297	0.0837	0.8160	0.6364
CR_DEATH	70	0.0160	0.1216	0.4280	0.0844	1.0000	0.7797
ST_B_Cell_Antigen_Receptor	38	0.0165	0.2273	0.0723	0.0844	0.8343	0.4471
bcl2family_and_reg_network	23	0.0151	0.0910	0.5598	0.0848	1.0000	0.7716
etsPathway	16	0.0189	0.1507	0.5197	0.0914	1.0000	0.7596
ST_Interleukin_4_Pathway	24	0.0209	0.0494	0.1223	0.0983	1.0000	0.5248
41bbPathway	18	0.0224	0.1738	0.6183	0.1026	0.8216	0.7795
MAP00561_Glycerolipid_metabolism	43	0.0231	0.2073	0.0959	0.1031	1.0000	0.5208
DNA_DAMAGE_SIGNALLING	90	0.0237	0.0668	0.3086	0.1031	0.9670	0.7330
egfPathway	27	0.0244	0.1037	0.0081	0.1036	0.7353	0.1961
pitx2Pathway	16	0.0269	0.0308	0.0267	0.1114	0.8736	0.4185
MAP00640_Propanoate_metabolism	19	0.0300	0.4536	0.8990	0.1160	0.9760	0.8450
ngfPathway	19	0.0287	0.0039	0.0017	0.1161	0.4056	0.0889

tpoPathway	23	0.0298	0.3028	0.3860	0.1178	0.8589	0.7719
CR_PROTEIN_MOD	146	0.0412	0.1908	0.0534	0.1353	0.8384	0.4579
EMT_UP	53	0.0431	0.4062	0.7524	0.1364	0.9723	0.8005
tumor_supressor	22	0.0384	0.2033	0.2490	0.1364	0.8132	0.6557
PROLIF_GENES	358	0.0401	0.0607	0.4379	0.1368	1.0000	0.7766
rasPathway	22	0.0409	0.0007	0.0017	0.1369	0.1092	0.0771
cxcr4Pathway	23	0.0370	0.0535	0.1223	0.1370	0.8346	0.5333
ctlPathway	15	0.0504	0.1986	0.3283	0.1370	1.0000	0.7594
nktPathway	27	0.0513	0.0650	0.1192	0.1373	0.9880	0.5286
pgc1aPathway	22	0.0396	0.1027	0.5993	0.1378	0.7452	0.7738
no2il12Pathway	17	0.0381	0.0315	0.0339	0.1381	0.8705	0.3920
CR_REPAIR	39	0.0429	0.4800	0.8777	0.1382	1.0000	0.8551
amiPathway	22	0.0502	0.1092	0.4061	0.1386	1.0000	0.7716
cskPathway	22	0.0502	0.1092	0.4061	0.1386	1.0000	0.7716
atrbrcaPathway	19	0.0463	0.2952	0.7569	0.1389	0.8689	0.7990
SIG_IL4RECEPTOR_IN_B_LYPHOCYTES	26	0.0368	0.0408	0.3440	0.1392	0.8859	0.7624
il7Pathway	16	0.0490	0.2132	0.5891	0.1398	1.0000	0.7796
pdgfPathway	27	0.0482	0.1478	0.0376	0.1398	0.7951	0.3846
cytokinePathway	21	0.0548	0.0251	0.0629	0.1402	0.8478	0.4293
hcmvPathway	16	0.0477	0.0685	0.0553	0.1407	0.7633	0.4597
MAP00361_gamma_Hexachlorocyclohexane_degradation	26	0.0462	0.4648	0.7872	0.1410	0.9483	0.8116
ST_ADRENERGIC	35	0.0547	0.3334	0.3985	0.1421	0.8967	0.7794
mef2dPathway	19	0.0458	0.0852	0.3248	0.1423	0.6816	0.7580
il17Pathway	17	0.0540	0.1360	0.4135	0.1424	1.0000	0.7801
SIG_CD40PATHWAYMAP	34	0.0574	0.4698	0.6131	0.1447	0.9396	0.7841
CR_TRANSCRIPTION_FACTORS	67	0.0625	0.2413	0.8066	0.1554	0.9781	0.8127
ST_Phosphoinositide_3_Kinase_Pathway	32	0.0674	0.1506	0.0285	0.1607	0.7831	0.3995
CR_CELL_CYCLE	78	0.0667	0.1602	0.4422	0.1612	0.7934	0.7638
MAP00970_Aminoacyl_tRNA_biosynthesis	16	0.0658	0.3413	0.5864	0.1613	0.8948	0.7799
MAP00650_Butanoate_metabolism	19	0.0741	0.4547	0.7897	0.1632	0.9666	0.8111
human_mitoDB_6_2002	326	0.0734	0.4476	0.8839	0.1637	0.9719	0.8518
nkcellsPathway	18	0.0731	0.0479	0.0975	0.1652	0.9341	0.5084
MAP00020_Citrate_cycle_TCA_cycle	18	0.0724	0.2228	0.4027	0.1658	1.0000	0.7761
MAP00480_Glutathione_metabolism	16	0.0706	0.4509	0.6679	0.1660	0.9770	0.7861
HTERT_DOWN	64	0.0716	0.0844	0.2132	0.1661	1.0000	0.6373
ccr3Pathway	22	0.0774	0.0394	0.1915	0.1663	0.8781	0.6138

DOWNREG_BY_HOXA9	28	0.0769	0.3795	0.5746	0.1673	0.9861	0.7838
lairPathway	15	0.0799	0.0687	0.0493	0.1675	0.9493	0.4375
chrebpPathway	17	0.0792	0.1321	0.4849	0.1681	1.0000	0.7499
nthiPathway	21	0.0820	0.2388	0.5235	0.1699	0.9810	0.7609
igf1Pathway	20	0.0896	0.0258	0.0039	0.1834	1.0000	0.1305
rac1Pathway	22	0.0969	0.0356	0.1856	0.1961	0.9256	0.6250
spryPathway	16	0.0983	0.0510	0.1073	0.1966	0.8840	0.5386
